# The pivotal role of malic enzyme in enhancing oil accumulation in green microalga *Chlorella pyrenoidosa*

**DOI:** 10.1186/s12934-016-0519-2

**Published:** 2016-07-07

**Authors:** Jiao Xue, Lan Wang, Lin Zhang, Srinivasan Balamurugan, Da-Wei Li, Hao Zeng, Wei-Dong Yang, Jie-Sheng Liu, Hong-Ye Li

**Affiliations:** Key Laboratory of Eutrophication and Red Tide Prevention of Guangdong Higher Education Institute, College of Life Science and Technology, Jinan University, Guangzhou, 510632 China

**Keywords:** Malic enzyme, *Chlorella*, Lipid accumulation, Biofuel

## Abstract

**Background:**

The fast growing photosynthetic microalgae have been widely used in aquaculture, food, health, and biofuels. Recent findings in the diatom has proposed a pivotal role of NADP-malic enzyme in generation of NADPH as an important supply of reducing power for fatty acid biosynthesis. To test the lipogenic malic enzyme for fatty acid synthesis in green algae, here the malic enzyme gene *PtME* from the oleaginous diatom *Phaeodactylum tricornutum* was expressed in a representative green microalga *Chlorella pyrenoidosa*.

**Results:**

The engineered *C. pyrenoidosa* strain showed higher enzymatic activity of malic enzyme which subsequently promoted fatty acid synthesis. The neutral lipid content was significantly increased by up to 3.2-fold than wild type determined by Nile red staining, and total lipid content reached 40.9 % (dry cell weight). The engineered strain exhibited further lipid accumulation subjected to nitrogen deprivation condition. Upon nitrogen deprivation, engineered microalgae accumulated total lipid up to 58.7 % (dry cell weight), a 4.6-fold increase over the wild type cells under normal culture condition. At cellular level, increased volume and number of oil bodies were observed in the engineered microalgal cells.

**Conclusions:**

These findings suggested that malic enzyme is a pivotal regulator in lipid accumulation in green microalga *C. pyrenoidosa*, and presenting a breakthrough of generating ideal algal strains for algal nutrition and biofuels.

## Background

Renewable biofuels have been considered as promising alternative energy sources for fossil fuels due to their beneficial properties such as renewable, non-toxic, biodegradable and low greenhouse gas emission [1, 2]. The conventional biodiesel was primarily produced from waste cooking oil, animal fat, and vegetable oils. However, these resources are not promising because of expensive production costs, limited availability of feed stock and competitive with food crops [3, 4]. These factors have played a vital role in evolving interest in renewable energy from microbial sources. Considering oil content, fatty acid compositions and growth rates, few of the traditional lipid-accumulating microbes including fungi and yeast have high potential for biodiesel production [5]. Thus, it is necessitated to explore the potential feedstock for biofuel production. The exploitation of microalgae as feedstock for the biofuel production has opened up new broad prospects. Microalgae are the most promising feedstock for biofuels due to their higher energy yields, rapid growth rate, independent of weather conditions, non-requirement of agricultural land hence not compete with the food crops, and lower environmental impacts [3, 4]. Identification of an ideal microalgal strain with a combination of characteristics including fast growth rate, high oil content, strong resistance and suitable for large-scale cultivation is the prerequisite for the microalgal biofuel industry [6]. Several microalgae species such as *Phaeodactylum* *tricornutum*, *Scenedesmus*, *Dunaliella*, *Chlorella*, and *Nannochloropsis* can generate high lipid content and grow fast [7–11]. Among these microalgae species, green microalga *Chlorella pyrenoidosa* has attracted considerable attentions mainly due to their higher growth rate, accumulate high levels of triacylglycerol (TAG) and other valuable byproducts, CO_2_ fixation [12–14]. They can accumulate high content of lipids under environmental stresses such as nitrogen deprivation [15, 16]. However, it is difficult to employ those methods in the large scale biofuel production due to economic value, environmental effects and labor-consuming [17, 18]. Hence, development of ideal microalgae with high oil-content, rapid growth rate is the need of the hour.

As lipids are highly reduced metabolites, biosynthesis of TAG requires high amount of reducing power. In the whole fatty acid biosynthetic pathway, the provision of reducing power is considered to be the major metabolic requirements in the oil-rich organisms. The provision of reducing power (NADPH) for the reduction of acetyl groups into the growing acyl chain of fatty acids has been considered to be the key factor in fatty acid biosynthesis [19]. NADP-malic enzyme (ME; EC 1.1.1.40) catalyzes the oxidative decarboxylation of malate to pyruvate by the reduction of NADP into NADPH, thus providing the source of NADPH for fatty acid biosynthesis. ME has been reported to be a major provider of the reducing power NADPH required for the lipid biosynthesis in oleaginous fungi [20]. Overexpression of ME resulted in significant increase in lipid content in some yeasts and molds [21–23]. Heterologous expression of *Mucor circinelloides* NADP+-dependent ME in oleaginous yeast *Rhodotorula glutinis* resulted in a 2.0-fold increase in lipid production [22]. In our previous study, we reported that characterization of a malic enzyme (PtME) in *P. tricornutum* with upregulted transcription under nitrogen limitation and its overexpression significantly enhanced the lipid content [6, 11]. Due to the remarkable role of ME in supplying NADPH, it is of great interest to investigate its effect on fast-growing green microalga *C. pyrenoidosa.* In this study, we tested the overexpression of *PtME* in *C. pyrenoidosa* resulted in enhanced lipid accumulation and this report will shed light on the development of industrially potential oleaginous microalgal strains.

## Results

### Sequence analysis of PtME

**Fig. 1:**
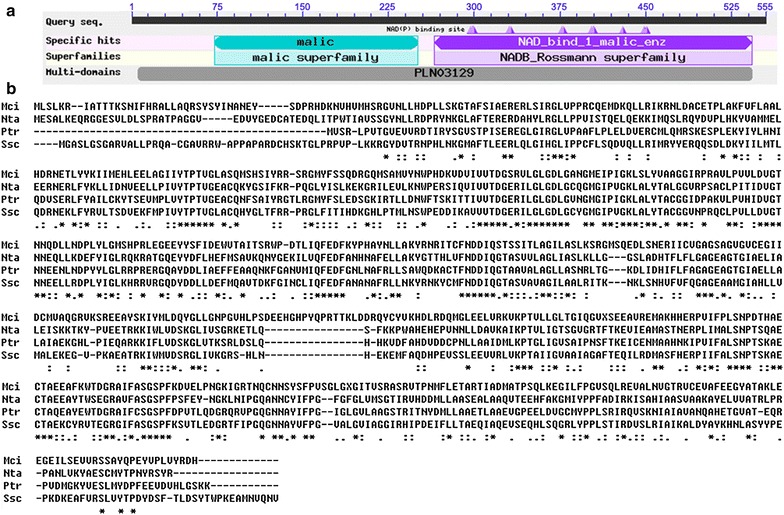
Sequence alignment of PtME with representative species based on BLAST. **a** Amino acid sequences of various species were analyzed with software MEGA5. *Mci* *Mucor circinelloides*, *Nta Nicotiana tabacum*, *Ptr Phaeodactylum tricornutum, Ssc* *Sus scrofa.* **b** The conserved domain “NAD_bind_1_malic_enz” indicates the “NAD(P) binding domain of malic enzyme (ME)”, and the conserved domain “malic” indicates the “Malic enzyme, N-terminal domain”

According to the sequence alignment, PtME amino acid sequence showed high homology with that in other species. The representative species in which ME had been functionally identified were aligned in Fig. 1. Accordingly, PtME possessed a conserved domain of “NAD(P) binding domain of malic enzyme (ME)” (amino acid 265–543) and a conserved domain of “Malic enzyme, N-terminal domain” (amino acid 73–251).

### Construction of expression vector and screening of engineered microalgae

**Fig. 2:**
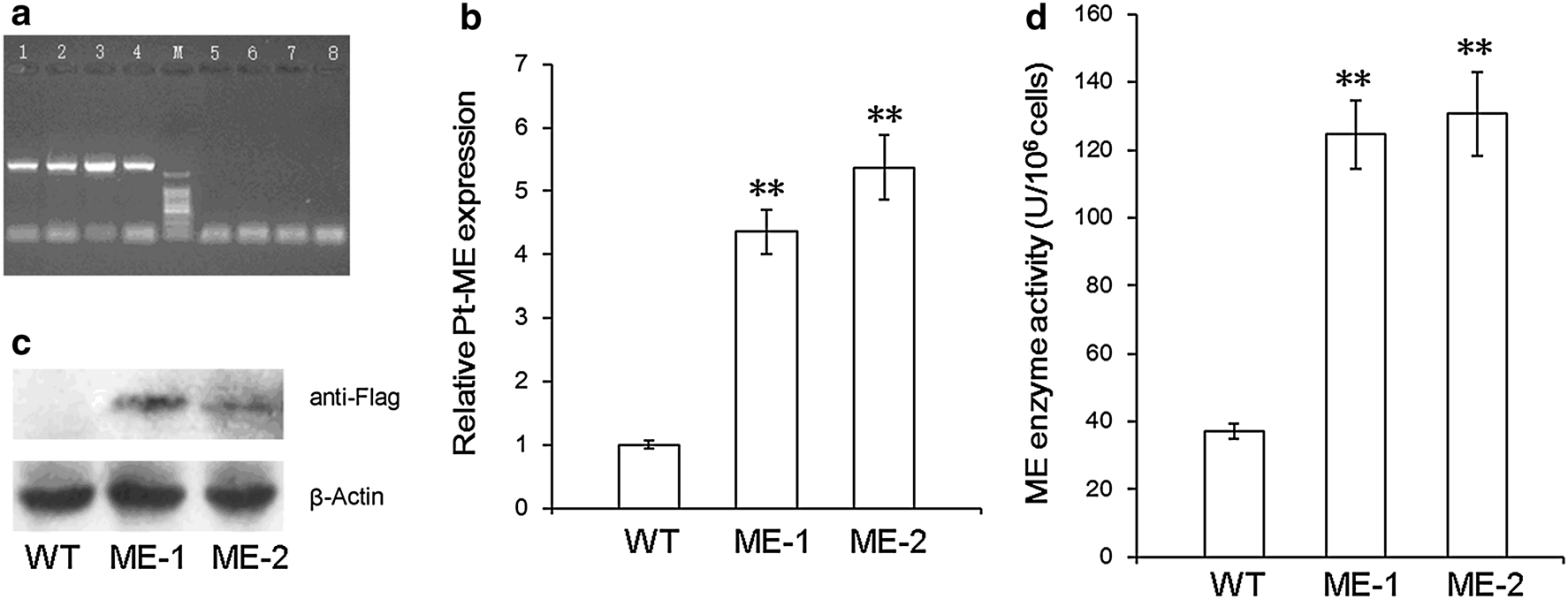
Expression of PtME in *C. pyrenoidosa.* **a** Single PCR analysis. *Lane* *1–4* transformed algae; *5–8*: wild type. **a** 1.68-kb band was detected in *lanes* *1–4* (*transformed lines*); while no such band was detected in *lanes* *5–8* (wild type). **b** Expression of PtME transcripts measured by qPCR; β-actin was used as internal reference gene. Significant difference between control and treatment groups is indicated at P < 0.05 (*) or P < 0.01 (**) level. Each value represents mean ± SD (n = 3). **c** PtME protein expression detected by western blotting with an anti-Flag antibody; beta-actin was used as internal reference protein. **d** Enzymatic activity of ME in transformed and wild type cells. **indicates a significant difference between wild type and engineered microalgae (P < 0.01)

PtME coding sequence was cloned into between fucoxanthin chlorophyll a/c binding protein (fcp) *fcpC* promoter and *fcpA* terminator of *P. tricornutum* in the transformation vector pHY-PtME. An Omega leader nucleotide motif was added upstream of *PtME* for enhancement of its translation. Engineered algal lines were selected under zeocin and cultured for five successive subculture cycles thereafter subjected to molecular characterization. Engineered cell lines were screened by single cell PCR to detect the integration of PtME expression cassettes by using the primers for the flanking region in the vector pHY-PtME. As expected 1.68-kb band using the primer sets which flanked the PtME sequence was found in engineered lines, but not in the wild type, demonstrating the successful integration of PtME cassette into transformed lines (Fig. 2a).

### Analysis of PtME expression in engineered microalgae

To determine the relative transcript abundance of *PtME* in engineered algal lines, qPCR analysis was performed in the cells after 10 days of subculture using β-actin as the internal reference gene. Both engineered lines ME-1 and ME-2 showed considerably high transcript abundanc of *PtME* (Fig. 2b). Furthermore, western blot analysis was conducted to determine the PtME protein expression using anti-flag antibody. This analysis revealed a specific protein band of 60.4-kDa which was in accordance with molecular weight of PtME in the engineered lines, but not present in the wild type cells, demonstrating successful expression of the PtME protein in the engineered lines (Fig. 2c). And malic enzyme activity turned out to be 36.9 ± 2.3 U/10^6^ cells in wild type and 124.6 ± 12.3 U/10^6^ cells in engineered lines, which excelled the wild type a lot (Fig. 2b). Consequently, it could be proposed that the enzymatic activity of malic enzyme was considerably enhanced by overexpression of PtME.

### Characterization of photosynthesis and growth of algal cells

**Fig. 3:**
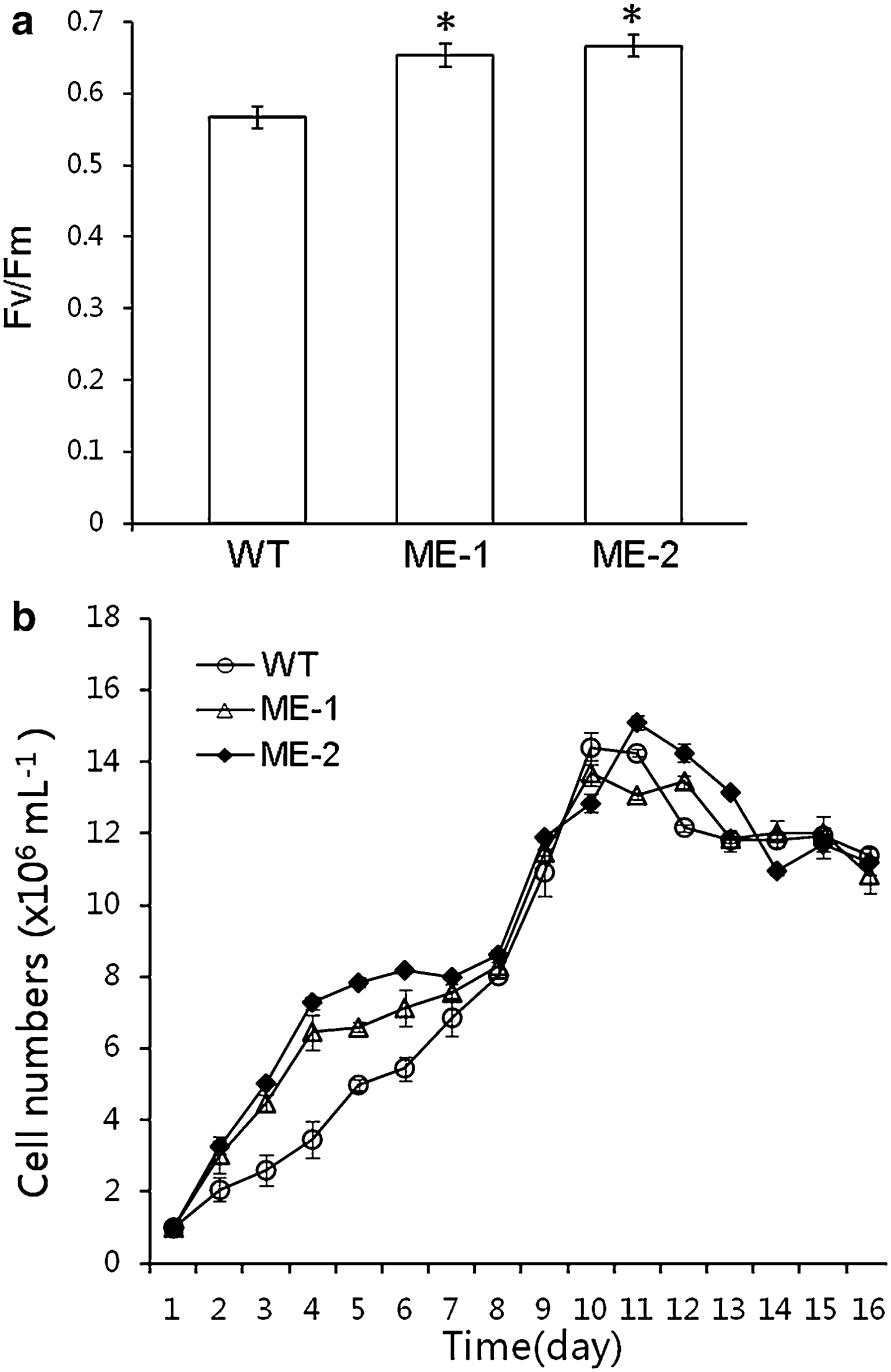
Photosynthesis performance and growth curve of *C. pyrenoidosa.* **a** Fv/Fm showing photosynthesis activity; **b** growth curves of engineered and wild type microalgae. Significant difference between control and treatment groups is indicated at P < 0.05 (*) or P < 0.01 (**) level. Each value represents mean ± SD (n = 3)

To analyse the photosynthetic efficiency of the cells, we measured the chlorophyll fluorescence parameter Fv/Fm (ratio of variable/maximum fluorescence) values for both the engineered and wild type cells during the early stationary phase (Fig. 3). The engineered lines ME-1 and ME-2 showed Fv/Fm values of 0.65 ± 0.01 and 0.67 ± 0.01, respectively, whereas wild type showed Fv/Fm value of 0.57. The results showed a markable increase in Fv/Fm value in engineered cells compared with wild type.

Growth curve was plotted for both engineered and wild type cells to determine the biomass accumulation. The results indicated that engineered cells exhibited a slightly faster growth rate till 8th day of the culture, thereafter both engineered and wild type cells showed almost similar growth rates during the stationary phase.

### Lipid accumulation in engineered microalgae

**Fig. 4:**
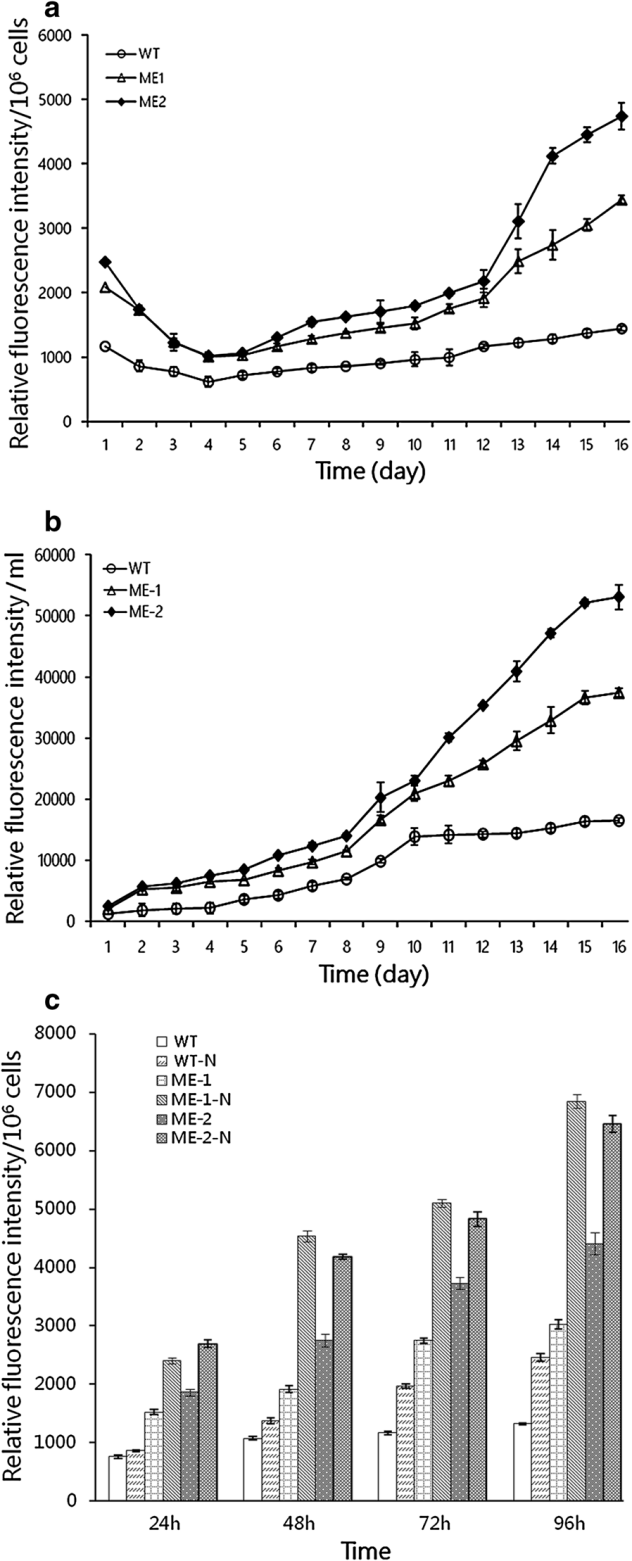
Lipid productivity in *C. pyrenoidosa.* **a** Neutral lipid content as per 10^6^ cells determined by Nile red staining. **b** Neutral lipid content as per culture volume. **c** Neutral lipid content under N-deprivation. Significant difference between control and treatment groups is indicated at P < 0.05 (*) or P < 0.01 (**) level. Each value represents mean ± SD (n = 3)

To correlate lipid accumulation with cell growth characteristics, the neutral lipid production over the growth period was examined by Nile red staining which is specific to neutral lipids. Until 10th day of subculture, neutral lipid content steadily increased in engineered lines, and the neutral lipid content per 10^6^ cells was higher than wild type, in particular, lines ME-1 and ME-2 showed an increase by 2.4- and 3.2-fold, respectively (Fig. 4a). As per the conventional gravimetry, total lipid content of wild type during the stationary phase was determined to be 12.7 %, while in engineered ME-1 and ME-2 lines, total lipid content attained up to 30.3 ± 1.2 and 40.9 ± 0.9 % respectively. As to the productivity per culture volume, neutral lipid content of unit volume in Lines ME-1 and ME-2 were 2.3- and 3.3-fold higher than the wild type (Fig. 4b).

Nitrogen (N) deprivation had been shown to enhance neutral lipid accumulation in certain microalgal species, hence, the capability of engineered microalgae to accumulate neutral lipids was also examined under N deprivation (−N) during the stationary phase (Fig. 4c). The relative neutral lipid content from the cells 48 and 96 h after −N was measured by Nile red staining. After 48 and 96 h of −N, the content of wild type was 1.3- and 1.7-fold higher, respectively. While engineered lines ME-1 was 4.2- and 4.6-fold higher, and ME-2 was 3.9- and 4.4-fold higher, respectively, compared to the wild type under normal condition, with up to 58.7 % of dry cell weight in the engineered microalgae. The content of ME-1-N and ME-2-N was 2.7- and 2.5-fold compared to WT-N, respectively, after 96 h of –N. These results suggested that N-deprivation could influence intracellular metabolic shifts, thus enhance the neutral lipid content of both wild type and engineered cells.

### Subcellular structure of engineered microalgae

**Fig. 5:**
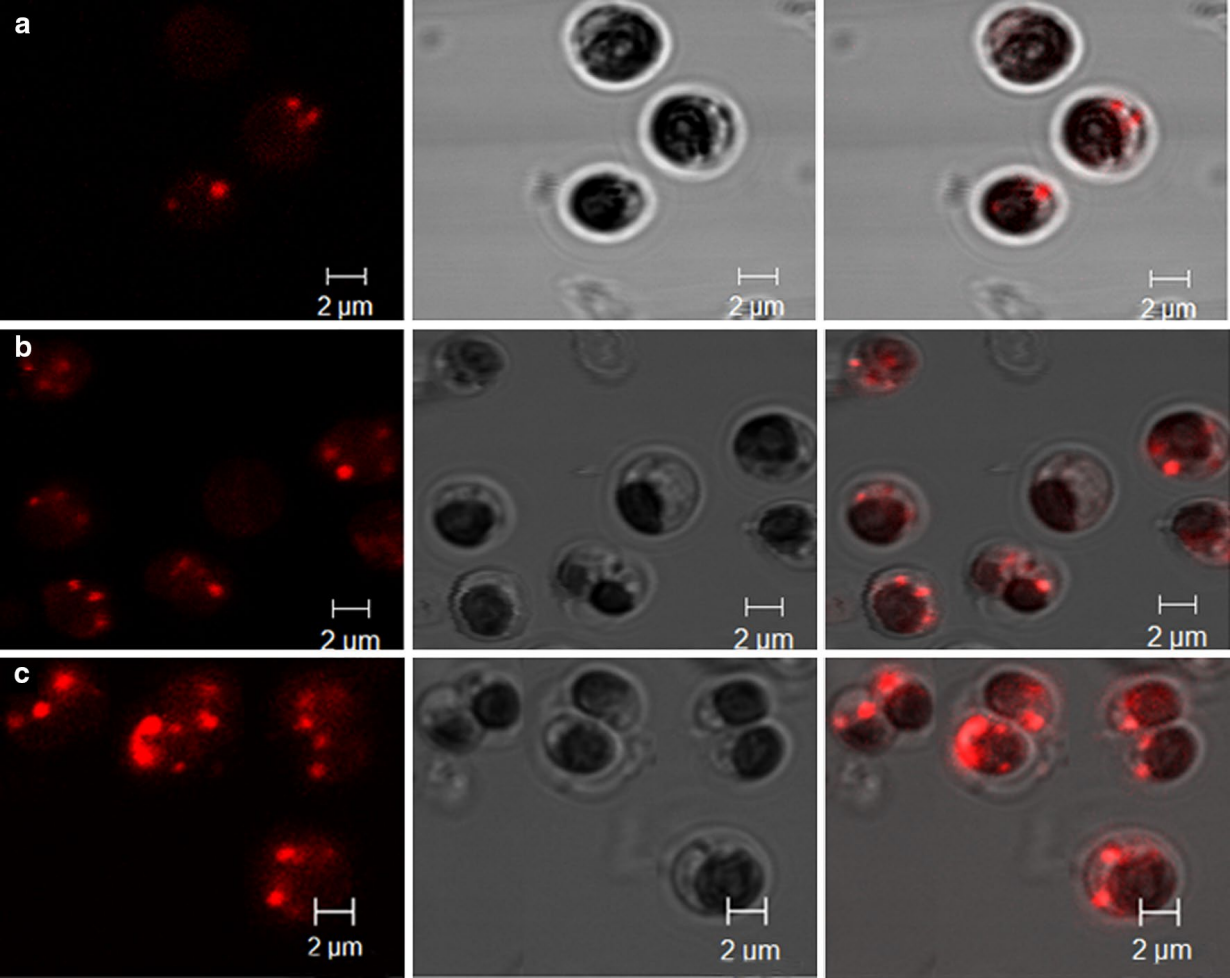
Morphology of microalgal cells and oil bodies. Microalgal cells were stained with *Nile red* and photographed under a laser-scanning confocal microscope. **a** wild type; **b**, **c** engineered cell lines ME-1 and ME-2. *Bar* 2 µm

To examine the alterations in the volume of oil bodies, microalgal cells in the late stationary phase, 15 days after subculture, were analyzed under scanning confocal microscopy (Fig. 5). The results revealed a concomitant increase in the volume of oil bodies in the engineered cell lines than the wild type. In contrast to the few oil bodies in wild type cells, engineered cells possessed apparently larger numbers of oil bodies. No other morphological changes could be observed. The results suggested that the total content of oil bodies was significantly enhanced in the engineered lines which could reflect the enhancement of neutral lipid content.

### Fatty acid composition analysis

In order to find out the influence of overexpressed *PtME* on fatty acid composition in the engineered and wild type cells, GC–MS analysis was carried out and the results are given in Table 1. It was notable that the composition of fatty acid in engineered lines was altered compared to that of wild type. The content of total saturated fatty acids (SUM SFAs) in the engineered lines ME-1 and ME-2 was decreased by 32.7 and 22.2 % respectively than wild type. Particularly, proportion of C17:0 and C18:0 in ME-1 showed 6.56- and 2.83-fold less, respectively than wild type and other SFA’s proportion was also appeared to decline significantly. On the other hand, total polyunsaturated fatty acids (SUM PUFAs) of transformed line increased by 34.0 %. The content of C16:3 and C18:3 in the engineered lines were increased 68.5 and 42.9 %, respectively compared to wild type, whereas the proportion of C16:2 showed a remarkable decline. Among the monounsaturated fatty acid, the proportion of C16:1 in the engineered lines was about 6.1-fold higher than wild type. This analysis depicted the role of PtME in influencing the fatty acid composition besides regulating lipogenesis.

**Table 1:** Fatty acid compositions (%) of *C*. *pyrenoidosa* cells

Composition	Wild type	ME-1	ME-2
C14:0	0.3 ± 0.03	0.18 ± 0.01	0.21 ± 0.01
C15:0	0.37 ± 0.04	0.17 ± 0.02	0.21 ± 0.03
C16:0	23.97 ± 2.46	19.2 ± 0.42	21.49 ± 1.15
C17:0	0.59 ± 0.11	0.09 ± 0.01	0.08 ± 0.01
C18:0	8.36 ± 1.69	2.95 ± 0.25	4.21 ± 0.19
SUM SFAs	33.59 ± 4.83	22.59 ± 0.71	26.2 ± 1.39
C16:1	0.42 ± 0.07	2.4 ± 0.23	2.54 ± 0.39
C18:1	1.18 ± 0.13	1.17 ± 0.37	1.15 ± 0.18
SUM MUFAs	1.6 ± 0.20	3.57 ± 0.60	3.69 ± 0.57
C16:2	1.89 ± 0.48	0.39 ± 0.04	0.04 ± 0.01
C16:3	8.24 ± 0.02	13.88 ± 0.52	13.88 ± 0.61
C18:2	12.29 ± 3.35	14.08 ± 1.43	12.85 ± 2.78
C18:3	18.9 ± 0.26	27.01 ± 2.69	26.27 ± 1.82
SUM PUFAs	41.32 ± 4.11	55.36 ± 5.68	53.04 ± 5.22

## Discussion

Provision of reducing power in the form of NAD(P)H is pivotal for various cell processes and fatty acid biosynthesis [24]. Previous study documented the enhanced fatty acid content by supply of exogenous energy and reducing power in photosynthetic organisms [25]. In the present study, we successfully overexpressed ME from the oleaginous diatom *P. tricornutum* in *C. pyrenoidosa* which showed the feasibility of developing oil-rich microalgae.

As the transformation efficiency was strongly impacted by the promoter, we also attempted to evaluate heterologous fucoxanthin chlorophyll a/c binding protein (fcp) *fcpC* promoter of diatom *P. tricornutum* for transgene expression in green alga *C. pyrenoidosa.* Engineered *C. pyrenoidosa* with high level expression of introduced ME was successfully generated. In addition, our previous data have shown that the *fcpC* promoter enhanced the expression of transgenes in diatom and green algae [26, 27]. On the other hand, attempt to introduce transgene under such diatom *fcpC* promoter in heterokont *Nannochloropsis oceanica* was unsuccessful (data not shown). These findings revealed the characteristics of *C. pyrenoidosa* to express transgenes under the heterologous diatom promoters.

We correlated the cell growth characteristics with lipid accumulation rate in engineered lines as well as wild type. The integration of the introduced genes in the electroporated host genome occurs randomly, which led to the various phenotypic characteristic of engineered lines. This analysis showed that during the cell division phase, i.e., until 10th day, cell concentration gradually increased and lipid accumulation rate was slower than afterwards. Lipid content was found to be increased rapidly after 8th day, once cells started to reach the stationary phase. It suggests that high cell division during log phase might affect lipid accumulation. Both the engineered cell lines (ME-1 and ME-2) accumulated high lipid content than the wild type which imply the functional role of overexpressed ME in lipogenesis. The introduced ME showed high transcript abundance in engineered lines as revealed by qPCR. Overexpression of mitochondrial ME in the *M. alpine* showed higher expression level of ME gene resulted in higher enzyme activity thus enhanced the fungal arachidonic acid [21]. The engineered *M alpine* cell lines showed 3.0-fold higher expression levels than the wild type cells [21]. In this study, the higher transcript abundance of ME consequently resulted in higher ME protein expression and enzymatic activity in the engineered lines.

Nitrogen deprivation is one of the conditions employed to accumulate lipid in the oleaginous microbes. We achieved the enhanced lipid accumulation in the engineered *P. tricornutum* overexpressing malic enzyme under nitrogen deprived condition [11]. In this study, engineered *Chlorella* overexpressing ME-1 and ME-2 with enhanced lipid accumulation was further subjected to nitrogen deprivation, which resulted in additional lipid accumulation. Under nitrogen limited conditions, the increased carbon flux for the lipid biosynthetic pathway resulted in the enhanced lipid content [6]. The two most important requirements during the fatty acid biosynthesis in the oleaginous microbes are the carbon flux pathway and provision of NADPH [21]. Thus, it may be conjectured that the ME overexpressed lines under nitrogen deprivation conditions resulted in the lipid accumulation to its maximum capability. Similarly, Xiong et al. [28] reported oil yield of 58.4 % DCW in *Chlorella protothecoides* sp. 0710 by combining autotrophic and heterotrophic growth.

ME plays a crucial role by providing NAD(P)H for fatty acid accumulation and desaturation [11]. In this study, GC–MS analysis revealed that total fatty acid composition was altered in the engineered lines compared to wild type cells. Overexpression of ME from the oleaginous fungus *Mortierella alpina* led to substantial increases in ME activity and lipid contents in the *M. circinelloides* strain R7B [23] and in *Rhodotorula glutinis* as well [22]. Meng et al. [29] reported that co-expression of ME and acetyl-CoA carboxylase in *Escherichia coli* resulted in 5.6-fold increase in fatty acid production. In this study, content of total MUFAs and PUFAs was increased by up to 130.6 and 34.0 %, respectively, while total saturated fatty acid content was decreased by more than 22.2 %, compared to wild type. Unsaturated fatty acid accumulation in the cells is considered to be an ideal characteristic of the feedstock to be employed for biofuel production [22]. Moreover, increased level of MUFA was desirable for biofuel production with improved quality [30]. Overexpression of mitochondrial malic enzyme in *M. alpine* increased the content of unsaturated fatty acids [21]. *M. circinelloides* overexpressing ME showed 2.5-fold increased lipid content and increase in unsaturation of accumulated fatty acids [23]. *Rhodotorula glutins* overexpressing *McME* resulted in altered fatty acid composition [22]. The provision of reducing equivalent in the form of NADPH is known to be the inevitable element in fatty acid synthesis. Fatty acid synthase catalyzes the synthesis and elongation of C16 fatty acid, which requires NAPDH as reducing equivalent. Similarly, fatty acid desaturase also catalyze the NADPH dependent conversion of saturated fatty acid to unsaturated fatty acids. The overall alterations in the fatty acid composition observed in this study could be due to the upregulation of the NADPH dependent enzyme activities caused by accelerated NADPH production.

In conclusion, we successfully generated engineered *C. pyrenoidosa* overexpressing PtME with enhanced lipid accumulation and altered fatty acid composition. Our data provide a support for further clarification of the potential role of ME as provider of lipogenic NAD(P)H as reducing power supply for fatty acid biosynthesis. This report represents an important biotechnological breakthrough in the research and development of algal biofuels.

## Methods

### Strain and culture conditions

*Chlorella pyrenoidosa* was obtained from the Freshwater Algae Culture Collection, Institute of Hydrobiology, CAS, China (No: FACHB-28). The green algae were grown as batch cultures in BG11 medium. The BG11 medium was filter-sterilized through a 0.22 μm membrane. The green algae was grown at 25 °C in an artificial climate incubator provided with cool-white fluorescence light of 200 μmol photons m^−2^ s^−1^ under 12/12 h light/dark. Growth curve was determined by counting with Brightline hemocytometer using microscope every day.

### Cloning, vector construction and algal transformation

The full-length cDNA of *PtME* from *P. tricornutum* cloned into the expression vector pHY11 previously was employed for transformation in this study. In this expression vector pHY11, *PtME* was cloned under the control of fcpC promoter and fcpA terminator from fucoxanthin chlorophyll a/c binding protein genes of *P. tricornutum*. *C. pyrenoidosa* was electroporated with the expression vector using Bio-Rad GenePulserXcell apparatus (Bio-Rad, USA) as previously reported [11]. The electroporated algal cells were transferred into liquid medium and cultured at shaker at dark for 24 h. Afterwards, the transformed cells were plated onto selection medium fortified with 10 μg ml^−1^ zeocin. The growing transformed cells in the selection medium were cultured in liquid medium with chloramphenicol and subcultured every week.

### Molecular characterization of transformed microalgae

The transformation was confirmed by single-cell PCR using the primers Pt89F (5′-CCAACAACAACAAACAACAAACAAC-3′) and Pt92R (5′- GAAACCAAAGCGGAGTGACTGCAAC-3′) specific for the *PtME* cDNA as well as pHY-PtME backbone. Two transformed lines, ME-1 and ME-2 with relatively higher lipid content and growth rates were chosen for further analysis in biological triplication.

To quantify the relative transcript abundance of *PtME*, qRT-PCR was carried out in Boxin Co. (Guangzhou, China). *PtME* was amplified with primers PMEf (5′-TATGAATGGACCGATGGGCG-3′) and PtMEr (5′-TACATGCAACCGACGTCCAA-3′). β-actin with the primers ACT1f (5′-AGGCAAAGCGTGGTGTTCTTA-3′) and ACT1r (5′-TCTGGGGAGCCTCAGTCAATA-3′) was used as an internal control. The amplification of β-actin was used to normalize data.

Expression of PtME protein in the transformants was analyzed by Western blotting. Protein detection was performed using flag antibodies (Sigma, USA), and β-actin was used as internal control as per the described protocol [11]. Enzymatic activity of ME was measured by using a Malic enzyme activity colorimetric quantitative detection kit (Keming Co., Suzhou, China) according to manufacturer׳s instructions.

### Fluorometric and gravimetric determination of lipid content and fatty acid composition analysis

Cellular neutral lipid content in *C. pyrenoidosa* was determined by using Nile Red staining. Staining was performed in a 96-well microtitre plate in triplicates. Cells were treated with 20 % DMSO for 20 min at room temperature. To 3 mL aliquot of pretreated cell culture added with 30 μL of Nile red (0.1 mg mL^−1^ in acetone) in triplicates, mixed by rapid inversion and incubated in darkness for 20 min at room temperature. Fluorescence was recorded using a Hitachi F4600 microplate reader (Hitachi, Japan) at a wavelength of 485-nm excitation and 552-nm emission. For confocal microscopic observation, cells were stained with Nile red (0.1 mg/mL in acetone in a 1:100 ration) and incubated in darkness for 20 min. Stained cells were observed under a laser-scanning confocal microscope Zeiss LSM510meta (Zeiss, Germany) with excitation and emission wavelength of 488 nm and 505–550 nm, respectively. The total lipid content of algae was also measured by gravimetric analysis as per the reported protocol [11]. N-deprivation was further conducted to assess its role in lipid accumulation as previously described [11].

Lipids were extracted from the stationary phase culture (250 mL each) of engineered and wild type, and subsequently quantification of fatty acids was carried out by chromatography-mass spectrometry (GC–MS) and analyzed at the Guangdong Institute of Microbiology, China as per the described protocol [31].

### Characterization of photosynthesis

Chlorophyll fluorescence parameter Fv/Fm (ratio of variable/maximum fluorescence), an important indicator of photosynthetic performance and acclimation status [32], was analyzed to characterize microalgal photosynthesis. Algal culture was kept in darkness for at least 40 min and the chlorophyll fluorescence in the *C. pyrenoidosa* culture was measured with a TD-700 fluorometer (Turner Design, USA) following the manufacture’s manual.

## Abbreviations

GC–MS: gas chromatography mass spectroscopy
ME: malic enzyme
MUFA: monounsaturated fatty acid
PUFA: polyunsaturated fatty acid
SFA: saturated fatty acid
TAG: triglycerol
